# Differences in trait impulsivity do not bias the response to pharmacological drug challenge in the rat five-choice serial reaction time task

**DOI:** 10.1007/s00213-018-4836-5

**Published:** 2018-01-26

**Authors:** Rebecca L. Barlow, Jeffrey W. Dalley, Anton Pekcec

**Affiliations:** 1 Boehringer Ingelheim Pharma GmbH & Co. KG, Division Research Germany, Birkendorfer Strasse 65, 88397 Biberach an der Riss, Germany; 2 Department of Psychology, University of Cambridge, Downing St, Cambridge, CB2 3EB UK; 3 Department of Psychiatry, University of Cambridge, Downing Street, Cambridge, CB2 2QQ UK

**Keywords:** Trait impulsivity, Behavioral inhibition, 5-CSRTT, MK-801, Yohimbine, Cocaine

## Abstract

**Rationale:**

Maladaptive impulsivity is symptomatic of several neuropsychiatric disorders including schizophrenia, attention-deficit hyperactivity disorder (ADHD), and substance abuse disorders; paradigms designed to assess the underlying neurobiology of this behavior are essential for the discovery of novel therapeutic agents. Various models may be used to assess impulsivity as measured by the five-choice serial reaction time task (5-CSRTT), including variable inter-trial interval (ITI) sessions, the selection of extreme high and low impulsivity phenotypes from a large outbred population of rats, as well as pharmacological challenges.

**Objectives:**

The aim of this study is to evaluate if pharmacological challenge models for impulsivity are biased by underlying differences in impulsivity phenotype.

**Methods:**

Extreme high and low impulsivity phenotypes were selected in the 5-CSRTT, and dose-dependent effects of various pharmacological challenges, namely MK-801, yohimbine, and cocaine, were evaluated on task performance, specifically accuracy and premature responses.

**Results:**

All three compounds increased premature responding, while a decrease in attentional performance occurred following MK-801 and yohimbine administration. No differences in drug-induced impulsivity between rats selected for high or low impulsivity or in parameters indicative of attentional performance could be determined.

**Conclusions:**

Our findings indicate that different pharmacological challenges increase impulsivity on the 5-CSRTT, with modest effects on attention. These effects were not influenced by underlying differences in impulsivity phenotype, which is an important prerequisite to reliably use these challenge models to screen and profile compounds with putative anti-impulsive characteristics.

## Introduction

Impulsivity is a complex, multifaceted behavioral construct, characterized by the tendency to act prematurely and without foresight (Dalley et al. 2011). It can be observed behaviorally as impaired response inhibition or the inability to tolerate delayed rewards. Maladaptive impulsivity is symptomatic of several neuropsychiatric disorders including schizophrenia (Heerey et al. 2007; Kaladjian et al. 2011), attention-deficit hyperactivity disorder (ADHD) (Schachar et al. 1995; Winstanley et al. 2006), and substance abuse disorders (Ersche et al. 2010). Additionally, there is evidence that impulsivity could be regarded as an intermediate endophenotype; patients with substance abuse disorders show increased levels of impulsivity on self-report questionnaires compared with healthy controls, but of particular interest is that their non-drug-taking siblings also show increased levels of impulsivity (Ersche et al. 2010). As such, evaluating and understanding the underlying neural basis of this behavior is important to identify novel drugs for the effective treatment of these disorders.

Behavioral challenges are frequently used on the five-choice serial reaction time task (5-CSRTT) as a means of stratifying rats according to their performance. The flexibility inherent in the task allows the experimenter to manipulate the exact timing of the stimulus presentation: to make the waiting period, or inter-trial interval (ITI), short, long, or unpredictable (Bari et al. 2008). Such manipulations allow for the study of natural variation in behavior and how this is related to normal variation in neurotransmitter function, which may be a more translational approach; however, they also introduce a number of difficulties. The selection of extreme phenotypes on the 5-CSRTT using extended ITI sessions requires a large number of animals in order to gain an experimentally useful number of high and low impulsivity subjects (Bari et al. 2008; Dalley et al. 2007), thus making this approach impartible when aiming to determine the effects of drugs and establish the pharmacokinetic-pharmacodynamic relationship in rats with selected high and low impulsivity phenotypes. Additionally, variable ITI sessions require extended testing sessions in order to reach a statistically useful number of trials for each ITI. As such, pharmacologically induced challenges may represent a simpler model of maladaptive impulsivity, especially in the context of drug testing and/or modeling specific circuit malfunctions. There is a plethora of evidence that suggests maladaptive impulsivity may arise due to dysfunctional interactions within the frontostriatal circuit. Initially, it was thought that dysfunction in the inhibitory process was modulated via top-down control mechanisms at the level of the prefrontal cortex (Aron et al. 2003; Rieger et al. 2003). However, it is now known that dysfunction at the level of the basal ganglia and midbrain can also result in extreme impulsivity phenotypes. Patients with cortical and basal ganglia damage show impaired behavioral inhibition compared with healthy controls (Agnoli and Carli 2012; Dalley et al. 2011), and recent evidence shows dopamine dysfunction within the midbrain of impulsive individuals (Cole et al. 2013; Ray et al. 2012). It is therefore unsurprising that pharmacological agents that disrupt transmission within this circuitry can be used to induce impulsivity on a number of behavioral tasks.

Impulsive behavior is subject to modulation by a number of neurotransmitters and neuromodulators; the most widely examined include dopamine (DA), serotonin (5-HT), and noradrenaline (NA). Drugs that block the reuptake of DA and NA, such as methylphenidate or atomoxetine, show clinical efficacy in the treatment of ADHD. Recently, there has been an increase in studies examining the contribution of other neurotransmitters to the regulation of impulsive behavior. Systemic or directed administration of *N*-methyl-d-aspartate (NMDA) receptor antagonists, such as MK-801, phencyclidine (PCP), and 3-(2-carboxypiperazine-4-yl)propyl-1-phosphoric acid (CPP), enhance impulsivity on the 5-CSRTT (Fletcher et al. 2011; Greco et al. 2005; Mirjana et al. 2004).

In this paper, we confirm that a number of drugs that directly or indirectly affect dopaminergic neurotransmission within frontostriatal circuitry (MK-801, an NMDA receptor antagonist; yohimbine, an α2 adrenoceptor antagonist; and cocaine) can be used to induce impulsivity on the 5-CSRTT, and furthermore, that none of these pharmacological challenges are biased by underlying variations in trait impulsivity. We believe these data further highlight the advantages of a drug challenge approach in interrogating the underlying neurobiology of impulsive behavior, compared with behavioral challenges such as extended or variable ITI sessions.

## Materials and methods

### Subjects

Ninety-six male Lister hooded rats, weighing 200–250 g at the start of training, were obtained from Charles River (Sulzfeld, Germany) and assessed for performance on the 5-CSRTT. All rats were housed in groups of four with food and water initially available ad libitum. All rats were permitted at least 5 days acclimatization before training on the 5-CSRTT. Food restriction was initiated when body weight was ≥ 300 g. Body weight was then maintained at 80–85% of free-feeding weight. All experimental procedures were authorized by the Local Animal Care and Use Committee and carried out according to the local animal care guidelines, AAALAC regulations, and the USDA Animal Welfare Act.

### 5-CSRTT

Thirty-two five-choice operant chambers (Med Associates Inc., St. Albans, USA) enclosed in sound-attenuating, fan-ventilated cubicles were used, as described previously (Bari et al. 2008; Carli et al. 1983). Briefly, each chamber comprised five evenly spaced apertures (2.5 × 2.5 × 4 cm) containing an LED light set into a curved wall at the rear of the chamber. A centrally located food magazine was located on the opposite wall of the chamber, into which 45 mg of reward pellets could be delivered (Sandown Scientific). Infrared beams located at the entrance of each aperture and the food magazine allowed detection of nose pokes. Task parameters and data collection were controlled by Med Associates Inc. software (St. Albans, USA).

The 5-CSRTT training protocol has been described previously (Isherwood et al. 2015, 2017). Each training session comprised 100 discrete trials and lasted up to 30 min. At later training stages, 100 trials were normally completed within 20 min. Training sessions were initiated by the illumination of the house light and magazine light and the delivery of reward pellets. Collection of the reward initiated the first trial. A single trial comprised an ITI followed by the pseudo-random illumination of one of the five apertures for a fixed duration (stimulus duration; SD). Following stimulus detection, a nose poke to the corresponding aperture within a fixed time interval (limited hold; LH) was required for reward delivery. Premature responses made during the ITI, incorrect responses, and responses made outside the LH (an omission) were punished with a timeout (TO), where the house light was extinguished for 5 s.

Premature responding was calculated as a percentage of completed trials (correct + incorrect + omissions). A premature response was deemed an incomplete trial and reset the current trial. Percentage accuracy was defined as the number of correct responses divided by the sum of correct and incorrect responses. Perseveration was calculated as the number of additional responses made in the same aperture following a correct response. Percentage omissions were calculated in terms of the number of completed trials.

Animals were deemed to be trained when they completed ≥ 50 correct trials, with ≥ 70% accuracy, and ≤ 20% omissions with an SD of 0.7 s, an ITI of 5 s, and a LH of 5 s. At this stage, perseverative responses (additional responses made to the same aperture following a correct response) were also punished with a 5 s TO and loss of food reward. The range to reach the final stage of task acquisition was 26–38 sessions.

### Impulsivity screening

Screening for impulsivity consisted of three “challenge” training sessions where the ITI was extended to 7 s to increase the occurrence of premature responses (Dalley et al. 2007). Each challenge session was separated by four baseline training sessions, during which task parameters were restored to the training configuration (5 s ITI). The mean percentage of premature responses made by each rat across the challenge sessions was calculated. Those with a poor or unstable performance, or that did not complete 100 trials on 3 challenge sessions, were excluded from the study (*n* = 4).

All remaining 92 rats were ranked, from high to low impulsive, based on the mean percentage of premature responses. The upper and lower 15th centiles of premature responders were termed high impulsive (HI, *n* = 13) and low impulsive (LI, *n* = 12), respectively. The remaining rats were termed mid impulsive (MI, *n* = 67).

### Drugs

Drugs were administered according to a randomized Latin square design. (+)-MK-801 hydrogen maleate and cocaine hydrochloride were purchased from Sigma Aldrich (Germany), and yohimbine hydrochloride was purchased from TCI Deutschland GmbH (Eschborn, Germany). The dose range and pre-treatment time were selected based on previous experience (Isherwood et al. 2015), in-house pharmacokinetic data, and published literature (Fletcher et al. 2011). Since a fully automated data acquisition system was used, the experimenter was not blinded to treatment. MK-801 was administered via subcutaneous injection, while yohimbine and cocaine were administered intraperitoneally. All drugs were administered in a volume of 1 ml/kg in phosphate-buffered saline (pH 7.4) using the dose of 0, 0.03, 0.06, 0.1 mg/kg for the MK-801 study; 0, 0.5, 1, 1.5, 2 mg/kg for the yohimbine study; and 0, 5, 7.5, 10 mg/kg for the cocaine study. Drugs were administered 10 min before the start of the behavioral test with a 5-s ITI. Injections were given on a 3-day cycle, starting with a baseline retention session. On the following day, injections were given prior to testing, and on the third day animals were not tested and remained in their home cage. After testing of yohimbine, two low-impulsive rats were excluded from further drug testing and euthanized due to poor health status. After testing of cocaine but before testing of MK-801, another low-impulsive rat and one high-impulsive rat were excluded due to their health status.

### Data analysis

Data were analyzed using SPSS version 21 and GraphPad 6; the latter software was used to create the figures. Repeated measures analysis of variance (ANOVA) with appropriate within- and between-subject factors was used to assess behavioral data. In case of a violation of sphericity, as shown by significant main effects in Mauchly’s test of sphericity, the Greenhouse-Geisser correction to the degrees of freedom was used to correct the *p* values. Where significant main effects or interactions were indicated, post-hoc analysis using Dunnett’s test was performed. Statistical significance was set at *p* < 0.05.

## Results

### Selection of HI and LI rats

**Fig. 1:**
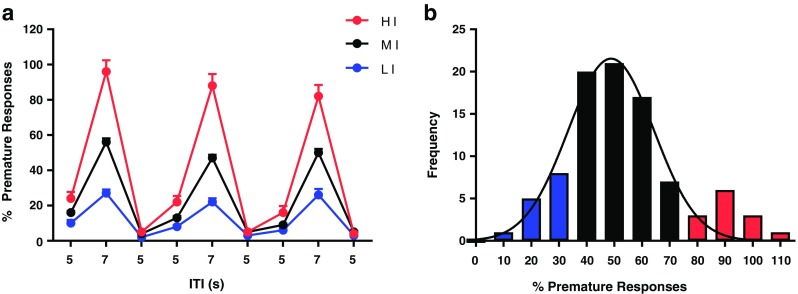
**a** Results from phenotypic screening, data presented as mean ± SD per daily screening session for high- (*n* = 13), mid- (*n* = 77), and low-impulsive (*n* = 12) rats. **b** Frequency distribution of premature responses, average across three 7 s ITI challenge sessions (*n* = 92), fitted with a unimodal Gaussian curve. Skewness = 0.54; kurtosis = 0.21; mean = 52.86; standard deviation = 20.17; median = 52.0; interquartile range = 21.75. *HI* high impulsive, *MI* mid impulsive, *LI* low impulsive

Rats were stratified according to their levels of trait impulsivity, as measured by premature responses across three 7 s ITI challenge sessions. In this cohort, the frequency of premature responses was significantly non-normal (*W*_(92)_ = 0.97, *p* < 0.05) and transformation of the data had no effect on the distribution (Fig. 1a). The rats were segregated into three groups according to their premature responding: (i) low impulsivity (lower 15%, *n* = 12); (ii) mid impulsivity (*n* = 67); and (iii) high impulsivity (upper 15%, *n* = 13) (Fig. 1b).

### Effect of yohimbine administration on 5-CSRTT performance in HI and LI rats

**Fig. 2:**
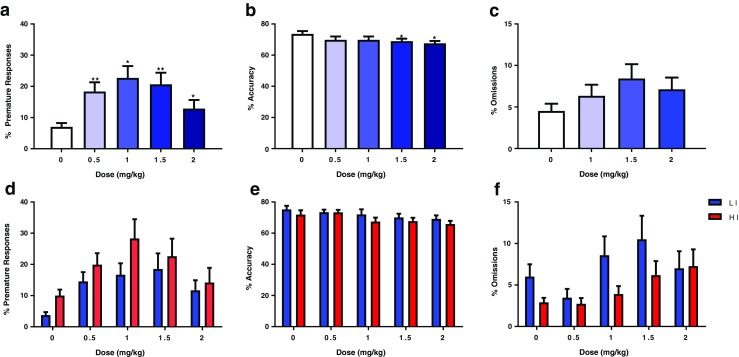
Effect of yohimbine (*n* = 25) on **a** percentage premature responses, **b** percentage accuracy, and **c** percentage omissions. Panels **d**–**f** depict the results when the group is split into low (*n* = 12) and high (*n* = 13) impulsive subjects. Data are mean ± SD. Asterisks denote a significant difference between the doses indicated: *p < 0.05 and **p < 0.01. *HI* high impulsive, *LI* low impulsive

Due to a software issue, no behavioral values were recorded for one low-impulsive subject at the 0.5 mg/kg dose level. For statistical analysis, the missing values were replaced with the group mean. Yohimbine produced a significant dose-dependent enhancement of impulsivity, as reflected by an increase in premature responding (*F*_(4,92)_ = 4.271, *p* < 0.014; Fig. 2a). Post-hoc tests revealed that all three doses of yohimbine significantly increased premature responding compared with vehicle treatment (0.5 mg/kg, *p* < 0.01; 1 mg/kg, *p* < 0.01; 1.5 mg/kg, *p* < 0.01; 2 mg/kg, *p* < 0.05). However, no significant dose × group interaction was observed (*F*_(4,92)_ = 2.044, *p* = 0.131; Fig. 2d). This was accompanied by a decrease in accuracy of performance (*F*_(4,92)_ = 5.736, *p* = 0.001; Fig. 2b) and an increase in omissions (*F*_(4,92)_ = 4.241, *p* = 0.009; Fig. 2c). As with premature responses, no significant dose × group interaction was observed for either behavioral measure (*F*_(4,92)_ = 0.354, *p* = 0.841; *F*_(4,92)_ = 1.65, *p* = 0.188; Fig. 2e, f). Yohimbine produced modest effects on response latencies and did not affect perseverative responses (Table 1).

**Table 1:** The effect of yohimbine, cocaine, and MK-801 of high- and low-impulsive rats on 5-CSRTT [correct latency, incorrect latency, reward latency, number of perseverative responses]

Drug/Response	Concentration (mg/kg)				
Yohimbine	0	0.5	1	1.5	2
HI					
Correct latency (s)	0.675 ± 0.092	0.645 ± 0.092	0.637 ± 0.094	0.662 ± 0.118	0.714 ± 0.125
Incorrect latency (s)	1.288 ± 0.456	1.301 ± 0.326	1.033 ± 0.189	0.944 ± 0.497*	1.364 ± 0.479
Reward latency (s)	1.198 ± 0.148	1.545 ± 0.873	1.220 ± 0.243	1.282 ± 0.228	1.375 ± 0.158**
Perseverative responses (n)	1.231 ± 1.092	3.462 ± 5.840	1.000 ± 1.414	1.538 ± 0.877	1.615 ± 1.557
LI					
Correct latency (s)	0.828 ± 0.163	0.751 ± 0.120	0.674 ± 0.090*	0.716 ± 0.122	0.704 ± 0.097*
Incorrect latency (s)	2.008 ± 0.531	1.578 ± 0.435	1.245 ± 0.380*	1.383 ± 0.503	1.331 ± 0.340*
Reward latency (s)	1.216 ± 0.100	1.313 ± 0.228	1.192 ± 0.073	1.197 ± 0.083	1.268 ± 0.112
Perseverative responses (n)	1.333 ± 1.435	1.364 ± 1.367	1.500 ± 1.508	1.417 ± 1.165	1.083 ± 1.505
Cocaine	0	5	7.5	10	
HI					
Correct latency (s)	0.645 ± 0.101	0.575 ± 0.104	0.563 ± 0.336	0.681 ± 0.334	
Incorrect latency (s)	1.372 ± 0.389	0.962 ± 0.390	1.095 ± 0.609	0.959 ± 0.722	
Reward latency (s)	1.365 ± 0.620	1.388 ± 0.344	1.238 ± 0.649	1.659 ± 0.851	
Perseverative responses (n)	1.846 ± 2.544	1.385 ± 1.609	2.000 ± 3.488	1.692 ± 2.323	
LI					
Correct latency (s)	0.788 ± 0.146	0.692 ± 0.157	0.717 ± 0.173	0.454 ± 0.264**	
Incorrect latency (s)	1.904 ± 0.484	1.562 ± 0.437	1.229 ± 0.893	0.986 ± 0.707*	
Reward latency (s)	1.323 ± 0.162	1.562 ± 0.437	1.485 ± 0.404	1.331 ± 0.918	
Perseverative responses (n)	1.300 ± 1.337	1.100 ± 1.370	0.500 ± 1.269	0.700 ± 0.823	
MK-801	0	0.01	0.03	0.1	
HI					
Correct latency (s)	0.671 ± 0.082	0.591 ± 0.091	0.739 ± 0.150	0.753 ± 0.152	
Incorrect latency (s)	1.297 ± 0.461	1.203 ± 0.452	1.285 ± 0.225	1.856 ± 0.513*	
Reward latency (s)	1.193 ± 0.098	1.085 ± 0.142**	1.332 ± 0.442	1.641 ± 0.421**	
Perseverative responses (n)	2.667 ± 1.614	2.083 ± 2.503	3.000 ± 2.594	4.083 ± 3.579	
LI					
Correct latency (s)	0.829 ± 0.119	0.674 ± 0.132**	0.730 ± 0.110	0.753 ± 0.314	
Incorrect latency (s)	1.969 ± 0.488	1.391 ± 0.543	1.379 ± 0.449*	1.810 ± 0.691	
Reward latency (s)	1.367 ± 0.346	1.144 ± 0.220**	1.207 ± 0.177	1.360 ± 0.572	
Perseverative responses (n)	1.556 ± 1.236	1.444 ± 1.590	3.222 ± 2.863	4.556 ± 2.744	

*5*-*CSRTT* five-choice serial reaction time task, *ANOVA* analysis of variance, *HI* high impulsive, *LI* low impulsive, *SD* standard deviation

Values represent mean ± SD. Repeated measures ANOVA, Dunnett’s post-hoc test (*p < 0.05, **p < 0.01 versus vehicle control)

### Effect of cocaine administration on 5-CSRTT performance in HI and LI rats

**Fig. 3:**
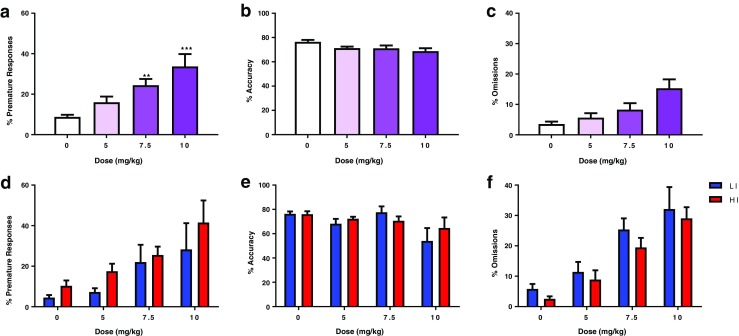
Effect of cocaine (*n* = 23) on **a** percentage premature responses, **b** percentage accuracy, and **c** percentage omissions. Panels **d**–**f** depict the results when the group is split into low (*n* = 10) and high (*n* = 13) impulsive subjects. Data are mean ± SD. Asterisks denote a significant difference between the doses indicated: **p < 0.01 and ***p < 0.001. *HI* high impulsive, *LI* low impulsive

Following yohimbine administration, two low-impulsive rats were euthanized due to poor health status, resulting in *n* = 10 low-impulsive rats and *n* = 13 high-impulsive rats for subsequent drug testing. Cocaine produced a significant dose-dependent enhancement of impulsivity, as reflected by an increase in premature responding (*F*_(3,63)_ = 5.763, *p* = 0.005; Fig. 3a). Post-hoc tests revealed that all three doses of cocaine significantly increased premature responding compared with vehicle treatment (5 mg/kg, *p* < 0.01; 7.5 mg/kg, *p* < 0.01; 10 mg/kg, *p* < 0.05). However, no significant dose × group interaction was observed (*F*_(3,63)_ = 0.473, *p* = 0.646; Fig. 3d). This was not accompanied by a decrease in accuracy (*F*_(3,63)_ = 0.868, *p* = 0.462; Fig. 3c) or an increase in omissions (*F*_(3,63)_ = 1.549, *p* = 0.211; Fig. 3c). Cocaine produced modest effects on response latencies and did not affect perseverative responses (Table 1).

### Effect of MK-801 administration on 5-CSRTT performance in HI and LI rats

**Fig. 4:**
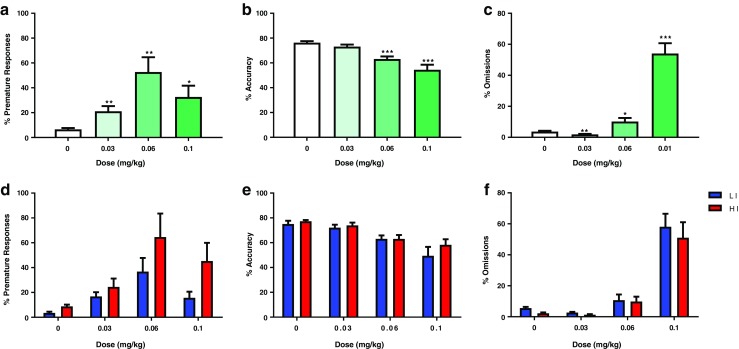
Effect of MK-801 (*n* = 21) on **a** percentage premature responses, **b** percentage accuracy, and **c** percentage omissions. Panels **d**–**f** depict the results when the group is split into low (*n* = 9) and high (*n* = 12) impulsive subjects. Data are mean ± SD. Asterisks denote a significant difference between the doses indicated: *p < 0.05, **p < 0.01, and ***p < 0.001. *HI* high impulsive, *LI* low impulsive

Following cocaine administration, a further low-impulsive rat and one high-impulsive rat were excluded due to their health status, resulting in *n* = 9 low-impulsive rats and *n* = 12 high-impulsive rats for subsequent drug testing. MK-801 produced a significant dose-dependent enhancement of impulsivity, as reflected by an increase in premature responding (*F*_(3,57)_ = 6.092, *p* < 0.007; Fig. 4a). Post-hoc tests revealed that all three doses of MK-801 significantly increased premature responding compared with vehicle treatment (0.03 mg/kg, *p* < 0.01; 0.06 mg/kg, *p* < 0.01; 0.1 mg/kg, *p* < 0.05). However, no significant dose × group interaction was observed (*F*_(3,57)_ = 0.73, *p* = 0.474; Fig. 4d). This was accompanied by a decrease in accuracy of performance (*F*_(3,57)_ = 17.474, *p* < 0.001; Fig. 4b) and an increase in omissions (*F*_(3,57)_ = 46.725, *p* < 0.001; Fig. 4c). As with premature responses, no significant dose × group interaction was observed for either behavioral measure (*F*_(3,57)_ = 0.673, *p* = 0.491; *F*_(3,57)_ = 0.154, *p* = 0.747; Fig. 4e, f). MK-801 produced modest effects on response latencies and did not affect perseverative responses (Table 1).

## Discussion

The 5-CSRTT is a widely used task to assess attention and aspects of behavioral inhibition. Here, we outline its use as a task of impulse control and detail the specific task manipulation that can be used to study both trait and induced impulsivity. Specifically, we emphasize the utility of pharmacological models in studying extreme impulsive behavior. We show that pharmacological challenges used to induce impulsivity, namely MK-801, yohimbine, and cocaine, are unaffected by underlying differences in trait impulsivity. All subjects showed the same dose response to the pharmacological challenge; as such, these models may be used without intrinsic and unknown impulsivity traits confounding the outcome of the challenge.

In humans and animals, yohimbine can induce transient anxiety, panic, stress, and mania-like and other hyper-arousal symptoms (Johnston and File 1989; Southwick et al. 1999; Stine et al. 2002) that are at least partially mediated directly by hypothalamic–pituitary–adrenal (HPA) axis activation or indirectly by engaging the extra-hypothalamic stress circuit and noradrenergic activity. It is conceivable that stress responses, including those evoked by yohimbine, affect impulse control. The first evidence for this assumption comes from an experimental medicine study assessing the effect of yohimbine on impulsivity (Swann et al. 2013). Specifically, a double-blind, placebo-controlled study in normal healthy volunteers investigated yohimbine’s effect on performance in the continuous performance task, the human analogue of the rodent 5-CSRTT. Yohimbine selectively increased the number and speed of impulsive responding but had no effect on any other task parameter. These effects were partially paralleled by an elevation of noradrenergic but not dopaminergic blood metabolites over time, possibly indicating engagement of the extra-hypothalamic stress circuit. Unfortunately, no self-reported measures of anxiety were included in this elegant study, which would have been interesting as patients with high baseline anxiety, as well as psychiatric patients, such as with post-traumatic stress disorder, are known to be particularly susceptible to the effects of yohimbine (Mattila et al. 1988; Southwick et al. 1999). Our data are generally in line with the observation of trait-dependent drug sensitivity, as HI rats have a numerically higher number of yohimbine-induced premature responses compared to LI rats. However, it is important to note that the extension of the upward shift in the dose-response curve in both groups of rats is not different, indicating that underlying trait impulsivity does not affect the general outcome of the drug challenge. However, it should be noted that we cannot fully exclude the possibility that lower doses than those employed in this study might differentially affect premature responding in HI and LI rats since yohimbine increased premature responding at the lowest doses tested.

Yohimbine reliably increases impulsivity in rats in a range of response-inhibition tasks (Mahoney et al. 2016; Schippers et al. 2016; Sun et al. 2010), some of which are conceptually analogue to the human Continuous Performance Task, suggesting that a yohimbine challenge can be considered as a tool for translational studies. Our data are not only generally in line with these reports but also extend these findings by demonstrating that yohimbine-induced impulsivity is not affected by underlying trait impulsivity. This is an important finding, considering that stratification on traits requires a high number of subjects, which is often not feasible, both in preclinical studies as well as exploratory clinical trials. Interestingly, no dissociation of responding between HI and LI animals has also been seen when using other noradrenergic agents, namely guanfacine and atomoxetine (Fernando et al. 2012).

High trait-like impulsivity is a well-known vulnerability marker/endophenotype for the abuse of stimulants and, potentially, other drugs. An elegant observational study from Ersche et al. (2010) determining trait impulsivity in siblings of chronic stimulant users evidenced this notion. Siblings who do not abuse stimulants reported significantly higher levels of trait impulsivity than age-matched control volunteers. Stimulant-dependent individuals reported even significantly higher levels of impulsivity than both their siblings and control volunteers, suggesting that impulsivity can be considered as a behavioral endophenotype and identifies subjects at risk for stimulant dependence that may be exacerbated by chronic drug exposure (Ersche et al. 2010). Additionally, high trait impulsivity predicts relapse in cocaine- and other stimulant-dependent individuals (Bosker et al. 2017; Moeller et al. 2001). In rats, high impulsivity predicts escalation of cocaine self-administration (Belin et al. 2008; Dalley et al. 2007), an increased propensity for relapse after abstinence, and compulsive drug taking (Belin et al. 2008; Economidou et al. 2009). High impulsivity on the 5-CSRTT is associated with reduced availability of dopamine D_2/3_ receptors in the ventral striatum (Caprioli et al. 2015; Dalley et al. 2007), alterations in dendritic spine density, and is selectively and causally determined by GABA-dependent mechanisms in the nucleus accumbens core (Caprioli et al. 2014).

Given the well-established link between dopaminergic signaling and impulsivity, it is not surprising that cocaine and other dopamine-elevating agents are frequently used in rodent choice reaction time tasks to increase impulsivity in order to study the effect of putative anti-impulsive drugs (Anastasio et al. 2011; Cunningham et al. 2013; Fletcher et al. 2011; Muschamp et al. 2014). Although dopaminergic drugs are frequently used as pharmacological challenges to evoke impulsivity, only one previous study determined potential differential effects in rats stratified for high and low baseline impulsivity. No dissociation of responding between HI and LI animals was identified when using different dopaminergic compounds, namely quinpirole, GBR12909, and methylphenidate but the D_2_ receptor agonist sumanirole selectively decreased premature responding in HI rats (Fernando et al. 2012). In contrast, baseline-dependent effects of methylphenidate have been described by Blondeau and Dellu-Hagedorn (2007). Using a cluster analysis approach, rats trained in the 5-CSRTT were separated according to their baseline performance into four distinct subgroups: efficient, middle, inattentive, and inattentive–impulsive rats. Methylphenidate significantly increased premature responses in middle, inattentive, and inattentive–impulsive rats at highest dose tested, whereas there was only a trend for increased premature responses in the efficient subgroup. These data suggest that high-impulsive rats are more sensitive to methylphenidate. However, in this study, methylphenidate also had significant effects on response and magazine latencies indicating that its effects on premature responding were not behaviorally selective. Nevertheless, in the 5-choice continuous performance task, methylphenidate decreased premature responding in high-impulsive rats but increased premature responding in low-impulsive rats (Tomlinson et al. 2014), consistent with the reported baseline-dependent effects of methylphenidate on premature responding and D_2_ receptor availability in the striatum (Caprioli et al. 2015). These studies highlight the importance of selecting drugs for a pharmacological challenge that do not differentially affect responding in HI versus LI rats. This requirement is met by cocaine, as shown by the current data. Cocaine increases the number of premature responses in a dose-dependent manner without changing performance accuracy, although a trend for increased omissions and decreased response latencies was apparent.

Dysfunctional glutamatergic signaling has been associated with a number of neuropsychiatric disorders such as schizophrenia (Deakin et al. 1989; Goff and Coyle 2001; Konradi and Heckers 2003; Lindsley et al. 2006), in which impulse control deficits are prominent. In experimental animals, systemic and local infralimbic administration of the NMDA receptor antagonists MK-801, PCP, and CPP, and NR2B-selective antagonists have the common effect of increasing impulsivity in rodents (Agnoli and Carli 2012; Benn and Robinson 2014; Carli et al. 1983; Fletcher et al. 2011; Greco et al. 2005; Higgins et al. 2003; Higgins et al. 2016; Isherwood et al. 2015; Murphy et al. 2005; Paine et al. 2007). This effect is at least partially mediated on the level of the prefrontal cortex, as indicated by local infusion studies. The neural mechanisms responsible for the observed behavioral effects are unclear but may involve modulation of glutamate release in the prefrontal cortex. Microdialysis studies have shown that NMDA receptor antagonists cause excessive neuronal firing (Jackson et al. 2004; Lecourtier et al. 2007), leading to increased extracellular glutamate efflux in the prefrontal cortex of freely moving rats (Ceglia et al. 2004; Moghaddam et al. 1997; Moghaddam and Adams 1998). It has thus been hypothesized that altered glutamatergic tone in the prefrontal cortex may underpin changes in impulsivity following administration of an NMDA receptor antagonist (Isherwood et al. 2015). This hypothesis receives support from clinical studies indicating a positive correlation between elevated glutamate levels in the anterior cingulate cortex with ADHD symptomatology and especially hyperactivity and impulsivity symptoms (Bauer et al. 2016). It has not been assessed up to this point if differences in glutamatergic signaling might underlie individual differences in trait-like impulsivity. Previous studies from our group demonstrate that neither modulation of metabotropic glutamate receptor 4 (group III) nor metabotropic glutamate receptor 5 (group I) differentially affects premature responses in HI or LI rats (Isherwood et al. 2015; Isherwood et al. 2017). In this study, the effects of the NMDA receptor antagonist MK-801 also did not depend on trait-like baseline impulsivity but resulted in a dose-dependent increase in the number of premature responses, which was concomitant with a dose-dependent decrease in accuracy. However, it should be noted that we cannot fully exclude the possibility that lower doses than those employed in the study might differentially affect premature responding in HI and LI rats, as MK-801 increased premature responding at the lowest doses tested. Interestingly, at the highest dose tested, the number of premature responses appeared to drop in both groups of rats and was associated with a pronounced increase in omitted trials, and increased latencies to respond and collect reward in HI rats. At this higher dose, the NMDA receptor antagonist tends to impair general task performance, an effect which may relate to the sedative or locomotor-disrupting effects of this class of drugs at higher doses (Gilmour et al. 2009; Imre et al. 2006; Isherwood et al. 2015).

The data presented here add to the existing literature, which shows that specific pharmacological manipulations of impulsivity on the 5-CSRTT are unaffected by underlying baseline levels of impulsive responding. Additionally, no other behavioral variables (accuracy, omissions, etc.) were differentially affected by any of the drugs tested, adding further weight to the case that these drugs have no distinguishable effects on any aspects of performance in rats selected for extreme premature responding. As such, they can confidently be used as challenges to enhance premature responding in rats unselected for pre-existing extreme variation in impulsivity, saving time and allowing for smaller group sizes to be used. This is an important prerequisite to reliably use these challenge models to screen and profile compounds with putative anti-impulsive characteristics.
